# Using different methods to process forced expiratory volume in one second (FEV _1_) data can impact on the interpretation of FEV _1_ as an outcome measure to understand the performance of an adult cystic fibrosis centre: A retrospective chart review

**DOI:** 10.12688/f1000research.14981.2

**Published:** 2018-08-17

**Authors:** Zhe Hui Hoo, Muhaned S.A. El-Gheryani, Rachael Curley, Martin J. Wildman

**Affiliations:** 1 School of Health and Related Research (ScHARR), University of Sheffield, Sheffield, S1 4DP, UK; 2 Sheffield Adult Cystic Fibrosis Centre, Northern General Hospital NHS Trust, Sheffield, S5 7AU, UK

**Keywords:** Cystic fibrosis, epidemiology, patient outcome assessment, forced expiratory volume

## Abstract

**Background: **Forced expiratory volume in one second (FEV _1_) is an important cystic fibrosis (CF) prognostic marker and an established endpoint for CF clinical trials. FEV _1_ is also used in observation studies, e.g. to compare different centre’s outcomes. We wished to evaluate whether different methods of processing FEV _1_ data can impact on centre outcome.

**Methods: **This is a single-centre retrospective analysis of routinely collected data from 2013-2016 among 208 adults. Year-to-year %FEV _1_ change was calculated by subtracting best %FEV _1_ at Year 1 from Year 2 (i.e. negative values indicate fall in %FEV _1_), and compared using Friedman test. Three methods were used to process %FEV _1_ data. First, %FEV _1_ calculated with Knudson equation was extracted directly from spirometer machines. Second, FEV _1_ volume were extracted then converted to %FEV _1_ using clean height data and Knudson equation. Third, FEV _1_ volume were extracted then converted to %FEV _1_ using clean height data and GLI equation. In addition, year-to-year variation in %FEV _1_ calculated using GLI equation was adjusted for baseline %FEV _1_ to understand the impact of case-mix adjustment.

**Results: Y**ear-to-year fall in %FEV _1_ reduced with all three data processing methods but the magnitude of this change differed. Median change in %FEV _1_ for 2013-2014, 2014-2015 and 2015-2016 was –2.0, –1.0 and 0.0 respectively using %FEV _1_ in Knudson equation whereas the median change was –1.1, –0.9 and –0.3 respectively using %FEV _1_ in the GLI equation. A statistically significant p-value (0.016) was only obtained when using %FEV _1_ in Knudson equation extracted directly from spirometer machines.

**Conclusions: **Although the trend of reduced year-to-year fall in %FEV _1_ was robust, different data processing methods yielded varying results when year-to-year variation in %FEV _1_ was compared using a standard related group non-parametric statistical test. Observational studies with year-to-year variation in %FEV _1_ as an outcome measure should carefully consider and clearly specify the data processing methods used.

## Introduction

Cystic fibrosis (CF) is a multi-system genetic condition but the two main affected organs are lungs (resulting in recurrent infections and respiratory failure) and gastrointestinal tract (resulting in fat malabsorption and poor growth) ^1^. Median survival has improved to 45 years, in part because of improvement in care quality ^2^. An important quality improvement initiative is benchmarking, which involves identifying high-performing centres and the practices associated with outstanding performance ^3– 5^. Since forced expiratory volume in one second (FEV _1_) is an important CF prognostic marker ^6– 9^, it is often used as an outcome measure for benchmarking ^3– 5, 10^.

Different statistical methods of analysing FEV _1_ data can yield different results ^11^, but there is scant attention paid to the methods of processing FEV _1_ data. We previously reported a statistically significant reduction in year-to-year %FEV _1_ fall for our CF centre from 2013–2016 ^12^. We now set out to understand the impact of using different FEV _1_ data processing methods on our CF centre’s outcome.

## Methods

This is a single-centre retrospective analysis of routinely collected clinical data from 2013–2016. Regulatory approval for the analysis was obtained from NHS Health Research Authority (IRAS number 210313). All adults with CF diagnosed according to the UK CF Trust criteria aged ≥16 years were included, except those with lung transplantation or on ivacaftor. These treatments have transformative effects on %FEV _1_^13– 15^, thus may affect the interpretation of year-to-year variation in %FEV _1_.

Demographic data (age, gender, genotype, pancreatic status, CF related diabetes, *Pseudomonas aeruginosa* status), body mass index (BMI) and FEV _1_ data were collected by two investigators (HZH and RC / HZH and MEG) independently reviewing paper notes and electronic records. Where data from the two investigators differ, the original data from paper notes or electronic records were reviewed to by both investigators to ensure the accuracy of abstracted data. This process ensures the accuracy of abstracted data and helps avoid potential bias from inaccurate or inconsistent data collection ^16^. FEV _1_ data were processed with three different methods prior to analysis. First, %FEV _1_ readings (calculated with Knudson equation ^17^ and available in whole numbers) were directly extracted from spirometer machines. Second, FEV _1_ volumes (in litres, to two decimal places) were extracted and clean height data were used to calculate %FEV _1_ (as whole numbers) with Knudson equation ^17^. Third, FEV _1_ volumes (in litres, to two decimal places) were extracted and clean height data were used to calculate %FEV _1_ with GLI equation ^18^ using an Excel Macro (Microsoft Excel 2013).

Best %FEV _1_, i.e. the highest %FEV _1_ reading in a calendar year for each study subject was used for analysis since it is most reflective of the true baseline %FEV _1_^19^. Year-to-year %FEV _1_ change was calculated by subtracting best %FEV _1_ at Year 1 from Year 2 (i.e. negative values indicate fall in %FEV _1_ and positive values indicate increase in %FEV _1_). In addition to calculating year-to-year %FEV _1_ change using three different FEV _1_ data processing methods, %FEV _1_ change calculated with GLI equation was also adjusted for baseline %FEV _1_ using reference values from Epidemiologic Study of CF (ESCF) ^20^. The ESCF study found median %FEV _1_ change of –3%/year, –2%/year and –0.5%/year for baseline %FEV1 ≥100%, 40–99.9% and <40% respectively ^20^. Adjusted %FEV _1_ change was calculated by subtracting median ESCF %FEV _1_ change from actual %FEV _1_ change. Thus, an adjusted %FEV _1_ change >0 meant the subject’s year-to-year change in %FEV1 was less than expected (indicating better health outcome) whilst an adjusted %FEV _1_ change <0 meant the subject’s year-to-year change in %FEV _1_ was more than expected (indicating worse health outcome).

%FEV _1_ change from 2013–2014 to 2015–2016 calculated using different FEV _1_ data processing methods were compared using Friedman test. Bland-Altman analyses ^21^ were also used to compare year-to-year variation in FEV _1_ as calculated with Knudson equation against year-to-year variation in FEV _1_ as calculated with GLI equation, to understand the impact of using different reference equations. Analyses were performed using SPSS v24 (IBM Corp) and Prism v7 (GraphPad Software). P-value <0.05 was considered statistically significant.

## Results

This analysis included 208 adults, with 147 adults providing data for all four years. Overall, the cohort was ageing but baseline %FEV _1_ increased from 2014 onwards (see Table 1).

**Table 1.** Characteristics of study subjects from 2013 to 2016.

	2013	2014	2015	2016
Excluded Lung transplantation, n On ivacaftor, n	6 7	6 7	9 9	7 13
**Included**, n	**166**	**170**	**185**	**186**
Age in years, median (IQR)	25 (19 – 31)	26 (20 – 32)	27 (20 – 34)	27 (21 – 34)
Female, n (%)	76 (45.8)	80 (47.1)	87 (47.0)	90 (48.4)
Genotype status: ^¶^ ≥1 unknown mutation(s), n (%) ≥1 class IV-V mutation(s), n (%) Homozygous class I-III, n (%)	11 (6.6) 26 (15.7) 129 (77.7)	13 (7.6) 29 (17.1) 128 (75.3)	16 (8.6) 36 (19.5) 133 (71.9)	15 (8.1) 34 (18.3) 137 (73.7)
Pancreatic insufficient, ^†^ n (%)	137 (82.5)	135 (79.4)	142 (76.8)	145 (78.0)
CF related diabetes, ^‡^ n (%)	39 (23.5)	42 (24.7)	42 (22.7)	54 (29.0)
*P. aeruginosa* status: ^§^ No *P. aeruginosa*, n (%) Intermittent *P. aeruginosa*, n (%) Chronic *P. aeruginosa*, n (%)	60 (36.1) 37 (22.3) 69 (41.6)	57 (33.5) 36 (21.2) 77 (45.3)	74 (40.0) 31 (16.8) 80 (43.2)	78 (41.9) 29 (15.6) 79 (42.5)
BMI, median (IQR)	22.3 (19.7 – 24.6)	22.7 (20.0 – 25.0)	23.0 (20.3 – 26.0)	23.2 (20.4 – 26.0)
Best %FEV _1_, median (IQR)	78.7 (54.1 – 92.5)	76.6 (54.4 – 89.7)	77.8 (60.4 – 89.0)	78.5 (58.5 – 89.6)

^¶^ Genotype status as defined by international consensus ^22^. Homozygous class I-III mutations indicate ‘severe genotype’.

^†^ Pancreatic insufficiency was diagnosed by the clinical team on the basis of ≥2 faecal pancreatic elastase levels <200µg/g stool and symptoms consistent with maldigestion and malabsorption, in accordance to the UK Cystic Fibrosis (CF) Trust guideline.

^‡^ CF related diabetes was diagnosed by the clinical team on the basis of oral glucose tolerance test and continuous subcutaneous glucose monitoring results, in accordance to the UK CF Trust guideline.

^§^ *Pseudomonas aeruginosa* status was determined according to the Leeds criteria ^23^.

The %FEV _1_ increase was in part due to younger adults with higher %FEV _1_ transitioning from paediatric care because %FEV _1_ tended to decline from year to year (see Table 2). However, different year-to-year change in %FEV _1_ results were obtained with different FEV _1_ data processing methods. There was statistically significant reduction in year-to-year fall in %FEV _1_ using %FEV _1_ readings as recorded in spirometer machines ( *p*=0.016). Cleaning of height data and standardisation of %FEV _1_ calculation with Knudson equation ^17^ did not alter the magnitude of year-to-year variation in %FEV _1_, but the p-value was no longer statistically significant ( *p*=0.062). The use of GLI equation altered the magnitude of year-to-year variation in %FEV _1_ although the trend of reduced year-to-year fall in %FEV _1_ persisted ( *p*=0.135). Adjustment for baseline %FEV _1_ further increased the p-value ( *p*=0.210).

**Table 2.** Discrepancies in year-to-year %FEV _1_ variation with different methods of processing forced expiratory volume in one second (FEV _1_) data.

Methods of processing FEV _1_ data:	Change in %FEV _1_, median (IQR)			Friedman test p-values
	2013 to 2014 (n = 158)	2014 to 2015 (n = 162)	2015 to 2016 (n = 176)	
(1) %FEV _1_ (calculated with Knudson equation) extracted from spirometer machines used for analysis ^†^	–2.0 (–6.0 to 1.0)	–1.0 (–3.3 to 2.0)	0.0 (–3.0 to 2.0)	0.016
(2) FEV _1_ volume (in L) extracted and height data were cleaned, then %FEV _1_ calculated using Knudson equation ^‡^	–2.0 (–5.0 to 1.0)	–1.0 (–4.0 to 1.0)	0.0 (–3.8 to 2.0)	0.062
(3) FEV _1_ volume (in L) extracted and height data were cleaned, then %FEV _1_ calculated using GLI equation ^ϕ^	–1.1 (–4.6 to 1.5)	–0.9 (–3.2 to 1,5)	–0.3 (–2.9 to 1.8)	0.135
(4) FEV _1_ volume (in L) extracted and height data were cleaned, then %FEV _1_ calculated using GLI equation, then change %FEV _1_ adjusted for baseline %FEV _1_ using ESCF reference values ^§^	0.7 (–2.4 to 3.6)	1.1 (–1.4 to 3.5)	1.6 (–1.3 to 3.7)	0.210

ESCF - Epidemiologic Study of cystic fibrosis

^†^ The vast majority of the %FEV _1_ data were from spirometer machines at the Sheffield Adult cystic fibrosis (CF) centre, which were calculated with Knudson equation ^17^ in whole numbers. Some %FEV _1_ data were from spirometer machines at the Pulmonary Function Unit which operationalised the Knudson equation differently; by calculating age to one decimal place to determine the predicted FEV _1_. These spirometer machines also provided %FEV _1_ to two decimal places, but this was rounded to whole numbers for the purpose of analysis. These results were presented at the 2017 North American CF Conference and were published as an abstract in Pediatric Pulmonology ^12^.

^‡^ FEV _1_ volumes were available in litres to two decimal places from spirometer machines. Height data were also extracted to allow the calculation of predicted FEV _1_. This led us to uncover the inconsistency recording of height, which affected 30–40% of the study subjects and would have introduced erroneous variability to the %FEV _1_ because all equations for predicted %FEV _1_ are dependent on height. Height data were cleaned to weed out error. Where there was uncertainty regarding the height, the higher value was used to obtain a conservative estimate of %FEV _1_. To replicate calculation process of the spirometer machines at the Sheffield Adult CF centre, age was rounded down to a whole number and predicted FEV _1_ in volume were calculated to two decimal places using Knudson equation ^17^. This was used to derive the %FEV _1_, which was then rounded to whole numbers for the purpose of analysis.

^ϕ^ FEV _1_ and height data were extracted as above. %FEV _1_ was calculated using the GLI equation ^18^ using an Excel Macro available at the European Respiratory Society website.

^§^ %FEV _1_ calculated using the GLI equation ^18^ as described above, then adjusted for baseline %FEV _1_ as described in the ‘Methods’ section. An adjusted %FEV _1_ change of >0 meant the subject’s year-to-year fall in %FEV _1_ was less than expected for his / her baseline %FEV _1_, indicating better health outcomes.

**Figure 1.**
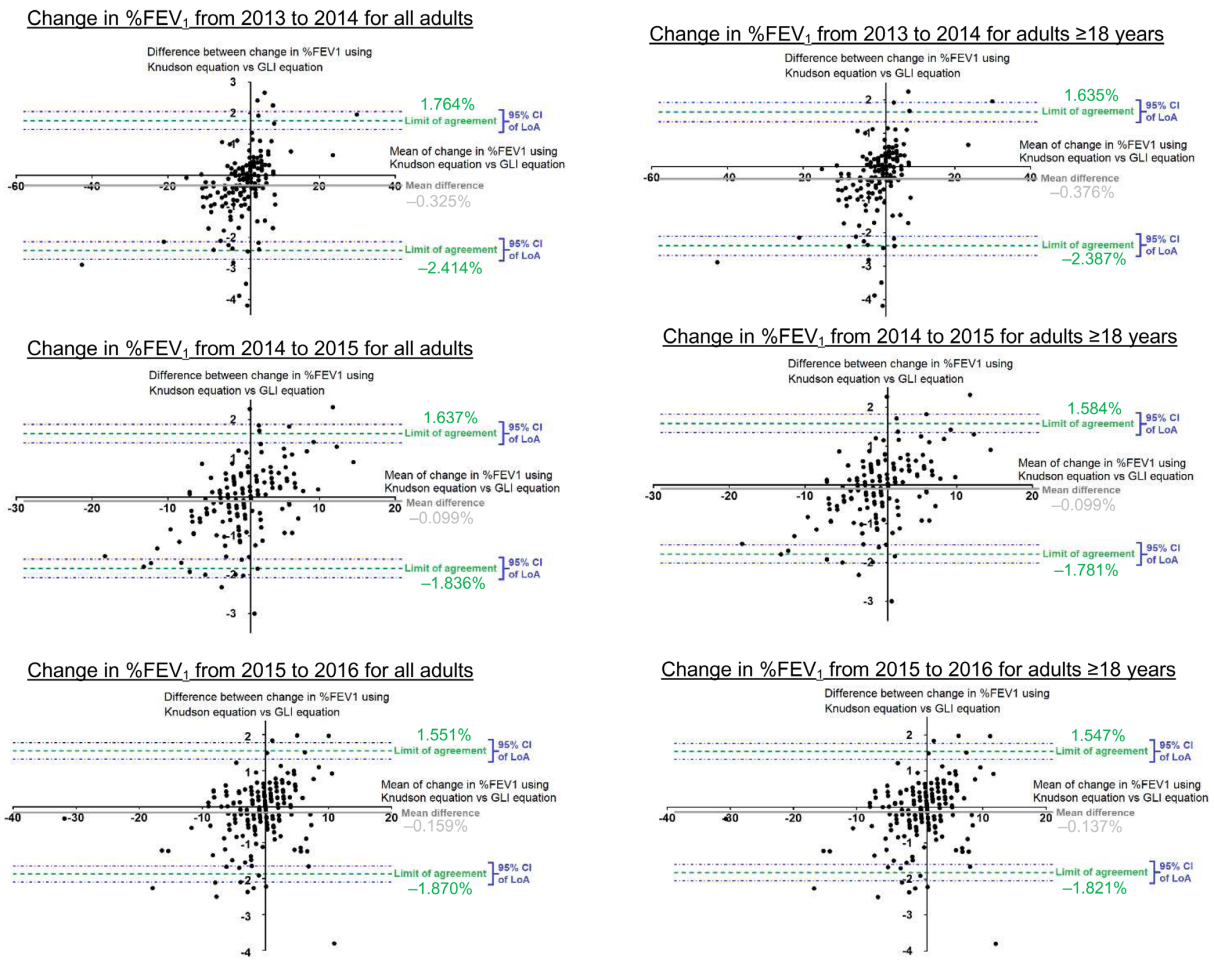
Bland-Altman plots comparing year-to-year variation in %FEV _1_ as calculated with Knudson equation (i.e. “Method 2” for processing FEV _1_ data according to Table 2) against year-to-year variation in %FEV _1_ as calculated with GLI equation (i.e. “Method 3” for processing FEV _1_ data according to Table 2).

Similar results were obtained when restricting the analyses to those aged ≥18 years (see Table 3). Bland-Altman analyses comparing year-to-year variation in %FEV _1_ calculated from clean FEV _1_ data using Knudson equation ^17^ vs year-to-year variation in %FEV _1_ calculated from clean FEV _1_ data using GLI equation ^18^ indicate the tendency for Knudson equation ^17^ to over-estimate the magnitude of year-to-year fall in %FEV _1_ by a mean difference of 0.1–0.4% (see Figure 1).

**Table 3.** Discrepancies in year-to-year %FEV _1_ variation with different methods of processing forced expiratory volume in one second (FEV _1_) data among adults aged ≥18 years.

Methods of processing FEV _1_ data:	Change in %FEV _1_, median (IQR)			Friedman test p-values
	2013 to 2014 (n = 147)	2014 to 2015 (n = 157)	2015 to 2016 (n = 172)	
(1) %FEV _1_ (calculated with Knudson equation) extracted from spirometer machines used for analysis	–2.0 (–6.0 to 1.0)	–1.0 (–3.0 to 2.0)	0.0 (–3.0 to 2.0)	0.016
(2) FEV _1_ volume (in L) extracted and height data were cleaned, then %FEV _1_ calculated using Knudson equation	–2.0 (–5.0 to 1.0)	–1.0 (–4.0 to 1.0)	0.0 (–3.8 to 2.0)	0.029
(3) FEV _1_ volume (in L) extracted and height data were cleaned, then %FEV _1_ calculated using GLI equation	–1.3 (–4.6 to 1.3)	–1.0 (–3.2 to 1.4)	–0.3 (–2.9 to 1.8)	0.090
(4) FEV _1_ volume (in L) extracted and height data were cleaned, then %FEV _1_calculated using GLI equation, then change %FEV _1_ adjusted for baseline %FEV _1_ using ESCF reference values	0.5 (–2.4 to 3.3)	1.0 (–1.4 to 3.4)	1.6 (–1.3 to 3.7)	0.149

Dataset 1. Sheffield forced expiratory volume in one second (FEV _1_) data http://dx.doi.org/10.5256/f1000research.14981.d205603
Copyright: © 2018 Hoo ZH et al. 2018 Data associated with the article are available under the terms of the Creative Commons Zero "No rights reserved" data waiver (CC0 1.0 Public domain dedication).

## Discussion

We demonstrated that different centre-level year-to-year variation in %FEV _1_ results were obtained using different FEV _1_ data processing methods. In particular, year-to-year fall in %FEV _1_ was smaller in magnitude when %FEV _1_ was calculated using GLI equation ^18^ instead of Knudson equation ^17^. This is in part due to the demographic of our centre which has a relatively young adult population. A previous study found a near-linear %FEV _1_ decline from childhood to adulthood with GLI equation, whereas there was accelerated %FEV _1_ decline during adolescence and young adulthood when %FEV _1_ was calculated with Knudson equation ^24^. One advantage of using the GLI equation, which is seamless across all ages, is that it improves the interpretation of %FEV _1_ decline ^24, 25^. Another advantage is that %FEV _1_ decline can be adjusted for baseline %FEV _1_ using ESCF reference values (since the ESCF values for %FEV _1_ decline were calculated using the GLI equation ^20^).

The limitation for all single-centre analysis is the potential lack of generalisability. Another limitation of our analysis is that the ESCF reference values used to adjust year-to-year variation in %FEV _1_ were derived using a cohort from around 15 years ago ^20^, and may not represent the current population. Our results nonetheless highlighted that year-to-year variation in %FEV _1_ can be extremely sensitive to the FEV _1_ data processing methods. This is one of the challenges of using year-to-year variation in %FEV _1_ to infer quality of care. Another challenge is that %FEV _1_ lacks sensitivity as an outcome measure. A recent sample size estimation using the UK CF registry data suggests that 273 adults per centre are needed to detect a 5% FEV _1_ difference at the 95% significance level ^26^. The sensitivity of measures used to detect variations in care quality is particularly pertinent to CF because a relatively small population is spread across many centres. Indeed, only 6/28 (21.4%) of all UK adult CF centres have ≥273 adults. That means process measures, e.g. medication adherence, is important to detect variations in quality of CF care. Mant & Hicks previous demonstrated that measuring processes of care proven in randomised controlled trials to reduce death allows detection of meaningful differences in care quality for myocardial infarction with just 75 cases, whereas 8179 cases would be needed if mortality was used as the quality indicator ^27^.

Given the limitations of FEV _1_ as an outcome measure in CF, results of centre comparisons based on FEV _1_ data should be carefully interpreted. Observational studies with year-to-year variation in %FEV _1_ as an outcome measure should carefully consider and clearly specify the data processing methods used.

## Ethical considerations

Regulatory approval for the analysis was obtained from NHS Health Research Authority (IRAS number 210313).

## Data availability

The data referenced by this article are under copyright with the following copyright statement: Copyright: © 2018 Hoo ZH et al.

Data associated with the article are available under the terms of the Creative Commons Zero "No rights reserved" data waiver (CC0 1.0 Public domain dedication). 

Dataset 1: Sheffield forced expiratory volume in one second (FEV _1_) data 10.5256/f1000research.14981.d205603 ^28^
