# Supplementary material for: Using different methods to process forced expiratory volume in one second (FEV 1) data can impact on the interpretation of FEV 1 as an outcome measure to understand the performance of an adult cystic fibrosis centre: A retrospective chart review
Source: F1000Res. 2018 Aug 17;7:691. Originally published 2018 Jun 1. [Version 2] doi: 10.12688/f1000research.14981.2 (PMC6213785; doi:10.12688/f1000research.14981.2)
Supplement: Peer review history

# Peer review: Referee response for version 2

**DOI:** 10.5256/f1000research.17436.r39259

**Published:** 2018-11-08

**Authors:** Clive Osmond

**Affiliations:** 1 MRC Lifecourse Epidemiology Unit, University ofSouthampton, Southampton, UK

Thank you for sending me this interesting note.  A few thoughts on the analysis from a statistician

1. It’s an interesting, though sobering, fact that between 30 and 40 percent of the machine-entered heights are incorrect. Normally the tendency would be for such errors to obscure, rather than generate, associations. This now-known, high error rate makes it less interesting to explore this section of the results.
2. What does a Friedman test measure? It’s a non-parametric version of a repeated measures one-way analysis of variance. Two issues are worth considering.It requires a complete table, so only those subjects with all four years of data may be included. Secondly it produces a three degree of freedom test, which is not very well directed to address the most likely question of interest. We might be most interested in detecting a smooth, linear trend over time. However just as much weight is being given to detect non-linear patterns such as curvature {low, high, high, low} and saw-tooth {low, high, low, high}. I don’t know how centres are compared officially. Comparison of neighbouring years’ data would be unstable. Also, using these non-linear components could be very misleading. I hope that the linear trend is used.
3. Are there any alternative analyses that would address these two issues?Certainly. To begin with, let’s use the original data for FEV1% and not worry about their normality. You could fit a mixed model to these data once you stack them in long format (“varstocases” in SPSS). This would enable you to use all data, not just data for those with a complete set. It would also enable you to extract a one degree of freedom test for trend across the four years.This should be a more powerful approach. I now see that the other referees refer to this as well, though I don’t agree that you need to have at least three observations per subject.  
4. If you are worried about the normality (though the published quartiles are not that alarming) then two alternatives would be (1) to find a normalising transformation that would apply to the stacked column of FEV1% values, or (2) to use a rank-based transformation (“Fisher-Yates”) available in SPSS as “rank y /normal into z.”
5. What might be the mathematics underlying any difference in slope obtained by the Knudson and GLI methods? I have tried to abstract the formulae used by Knudson and by GLIin deriving the predicted FEV1 that is used in the calculation of FEV1%. For a specific example I have chosen males (slightly more common in this study) aged 25 to 28 years (somewhere near the median age) with height of 175cm (just below mean UK adult height).  

The Knudson equation has a functional form

 FEV1 predicted = 5.1228 – 0.0292.age.

 FEV1% = FEV1/(FEV1 predicted) can then be differentiated to see how it varies with changes in age and FEV1

 However the GLI equivalent is given as point estimates from a Cole-Green LMS fitting procedure. The penalised cubic splines are not given, so no functional form is available.

 ​The table shows, just for this combination, how the predicted values compare.Those from Knudson are slightly lower and decrease slightly more rapidly with age.Such differences, and those from other combinations, will work together to determine how FEV1% might be expected to change with age and observed FEV.

I have read this submission. I believe that I have an appropriate level of expertise to confirm that it is of an acceptable scientific standard, however I have significant reservations, as outlined above.

# Peer review: Referee response for version 2

**DOI:** 10.5256/f1000research.17436.r37306

**Published:** 2018-10-31

**Authors:** Pierre-Régis Burgel

**Affiliations:** 1 Pulmonary Department and Adult CF CentreGroupe Hospitalier Cochin-Hotel Dieu, Paris Descartes University, Paris, France

No further comment

I have read this submission. I believe that I have an appropriate level of expertise to confirm that it is of an acceptable scientific standard.

# Peer review: Referee response for version 1

**DOI:** 10.5256/f1000research.16309.r34826

**Published:** 2018-07-17

**Authors:** Pierre-Régis Burgel

**Affiliations:** 1 Pulmonary Department and Adult CF CentreGroupe Hospitalier Cochin-Hotel Dieu, Paris Descartes University, Paris, France

The authors performed a retrospective analysis of FEV1% predicted data over 3 years in an adult CF center in the UK. They examined FEV1 decline from year to year by calculating variation in best FEV1 during two consecutive years and examined the impact of using data obtained using Knudson equation (directly extracted from the spirometer or recalculated with the appropriate height) vs. GLI equation. They also performed an adjustment using ESCF data.

 The authors concluded that trends in FEV1 decline were robust among methods, although the results were somewhat different using different methods/equations.

 The study has some interest in highlighting problems associated with these type of calculations, especially when used for benchmarking (as in the UK).

 I have the following comments for improvement:

1.  An important drawback of Knusdon equation is related to the change of FEV1 in the transition from pediactric to adults. This is why the GLI is nowadays often used in mixed pediatric/adult population. The authors used the UK definition of adults (over 16 years) and suggested that some of the difference in their results between Knudson and GLI data are due to the younger patients in this cohorts. I would be happier if the authors could perform a sensitivity analysis using only patients 18 years an over? This would miniminze the Knusdon/GLI age bias and would make these results more relevant to the adult centres outside of UK. Looking at Table 1, it seems that only a minority of patients were below 18 years.
2.  I think the word FEV1 decline is inappropriate in this manuscript. A year to year variation (even over 3 years) is not a decline. For calculating a decline, you would need multiple data points (at the very least 3 data points) and perform more complicated analyses (e.g., mixed model analysis). I would suggest to remove the word decline from the manuscript as the main goal of the authors did not appear to be FEV1 decline but mostly year to year FEV1 variation which is used for benchmarking in the UK.

I have read this submission. I believe that I have an appropriate level of expertise to confirm that it is of an acceptable scientific standard, however I have significant reservations, as outlined above.

# Response

**Published:** 2018-08-12

**Authors:** Zhe Hui Hoo

**Affiliations:** University of Sheffield, UK

We thank Prof Burgel for the review and we will iterate the manuscript taking into account the two very useful suggestions, i.e.

 1. we will perform a sensitivity analysis for the results in Table 2 using only adults aged 18 years and above

 2. we will replace the term "FEV1 decline" with "year-to-year FEV1 variation"

# Peer review: Referee response for version 1

**DOI:** 10.5256/f1000research.16309.r34828

**Published:** 2018-07-06

**Authors:** Edward McKone

**Affiliations:** 1 Department of Respiratory Medicine, St Vincent’s Hospital, Dublin, Ireland

FEV1 as a percent of predicted is widely used as an outcome measure in patients with cystic fibrosis and is one of the metrics used to compare centres or countries in benchmarking exercises.  This manuscript presents data showing that differences in data processing and the use of different reference equations used to estimate FEV1 as a percent predicted can result varying estimates of lung disease changes and potentially impact comparisons of centres/countries.

 The paper supports the standardization of FEV1 collection and reference equations which is currently in development by CF International Registries. It also highlights that different approaches to data collection can impact the interpretation of statistical analyses.

 Comments:

 Differences in FEV1 percent predicted using different equations is well known (Rosenfeld et al ^1^ and more recently in the cited UK/US comparison study). For this reason, the GLI have been recently accepted as the standard for most CF registries.

 Although year to year subtraction is a method of looking at longitudinal changes, regression methodology is preferable to analyse these changes, especially, as in this case, where you have 3 time points. This also allows to adjust for baseline factors such as lung disease severity.

 The method of adjustment for baseline Iung function is a bit crude. The medians subtracted are from a US population over 10 years ago and are likely to overestimate lung function decline in this population. In the Morgan et al, J Pediatr 2016 paper cited, the benefits of using this type of adjustment was shown using regression.

 Did their statistical approach factor in that these were repeated measures in the same patients?

 Bland & Altman plots comparing different reference equations could be considered.

 The results suggest that height inaccuracy is impacting the results.  As this is a single centre study, it is difficult to determine is this is a more universal problem.

I have read this submission. I believe that I have an appropriate level of expertise to confirm that it is of an acceptable scientific standard.

## References

[1] RosenfeldMPepeMSLongtonGEmersonJFitzSimmonsSMorganW: Effect of choice of reference equation on analysis of pulmonary function in cystic fibrosis patients. *Pediatr Pulmonol*.2001;31(3) :227-37 1127613610.1002/ppul.1033

# Response

**Published:** 2018-08-12

**Authors:** Zhe Hui Hoo

**Affiliations:** University of Sheffield, UK

We thank Prof McKone for the review and we will iterate the manuscript taking into account the suggestion to compare the different reference equations (Knudson vs GLI) using Bland-Altman analysis.

 We concur the GLI has been recently accepted as the standard for most CF registries.

 We concur that regression analyses is preferable to determine FEV1 decline. As recommended by Prof Burgel, we will replace the term "FEV1 decline" with "year-to-year FEV1 variation" in the revised manuscript.

 We concur that the method used to adjust year-to-year FEV1 variation for baseline FEV1 is crude. The displayed data from the ESCF paper is only presented according to the four FEV1 categories, hence our choice of adjustment method. Given the limited number of subjects within the Sheffield dataset, we felt is it is more appropriate to use reference values for suitably large datasets instead of simply calculating the predicted %FEV1 change using the Sheffield dataset. There are more recent reference values for FEV1 from the ECFSPR (Boëlle et al, 2012) and Canadian registry (Kim et al, 2018); however those papers do not provide reference values for year-to-year FEV1 variation.

 Our statistical method account for repeated FEV1 measures since:

 1. by using best FEV1, there is only x1 FEV1 reading per person per year

 2. only x1 FEV1 reading per person was used to calculate the year-to-year FEV1 variation 

 As mentioned in the discussion section, we concur that a single-centre study may not be generalisable. However, inaccurate data recording within routine datasets (e.g. CF registries) is unlikely to be an isolated problem. For example, the letter by Hartley et al (2016) in JCF revealed that 6% of the adults with CF at the Manchester Adult CF Centre had incorrect genotype data recorded in the UK CF registry.
